# Gastrointestinal Stromal Tumor as a Rare Cause of Abdominal Mass: A Case Report and Literature Review

**DOI:** 10.7759/cureus.14070

**Published:** 2021-03-23

**Authors:** Ahmed Baiomi, Hafsa Abbas, Balar Bhavna

**Affiliations:** 1 Internal Medicine, BronxCare Health System, New York, USA; 2 Gastroenterology, BronxCare Health System, New York, USA

**Keywords:** gastrointestinal stromal tumor (gist), stomach cancer, indeterminate abdominal mass, left sided abdominal pain

## Abstract

Gastrointestinal stromal tumors (GISTs) are soft tissue sarcomas that can arise from any part of the digestive system. GISTs represent 1% of gastric neoplasms. We report a rare case of a GIST tumor in a 59-year-old woman who presented with abdominal pain and weight loss. Computed tomography (CT) of the abdomen with intravenous contrast revealed a left upper quadrant abdominal mass and biopsy showed GIST. She was treated with imatinib to downsize the tumor prior to undergoing surgical resection of the tumor.

## Introduction

GISTs are one of the neoplasms originating from the gastrointestinal tract. They usually present as a subepithelial neoplasm and are often discovered during endoscopy done for various indications. They can originate from any part of the gastrointestinal tract [1]. We present a rare case of GIST where the patient presented with weight loss, abdominal pain and distension.

## Case presentation

**Figure 1:**
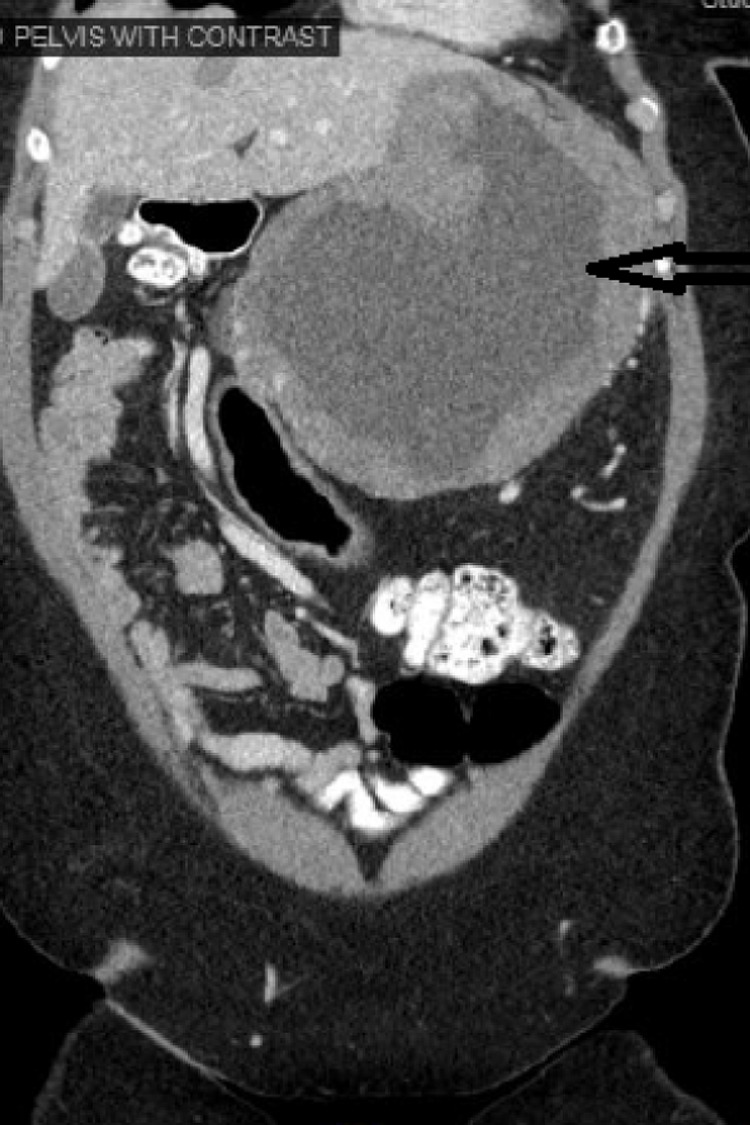
Computed Tomography of the abdomen and pelvis with coronal view Black arrow pointing to the mass

**Figure 2:**
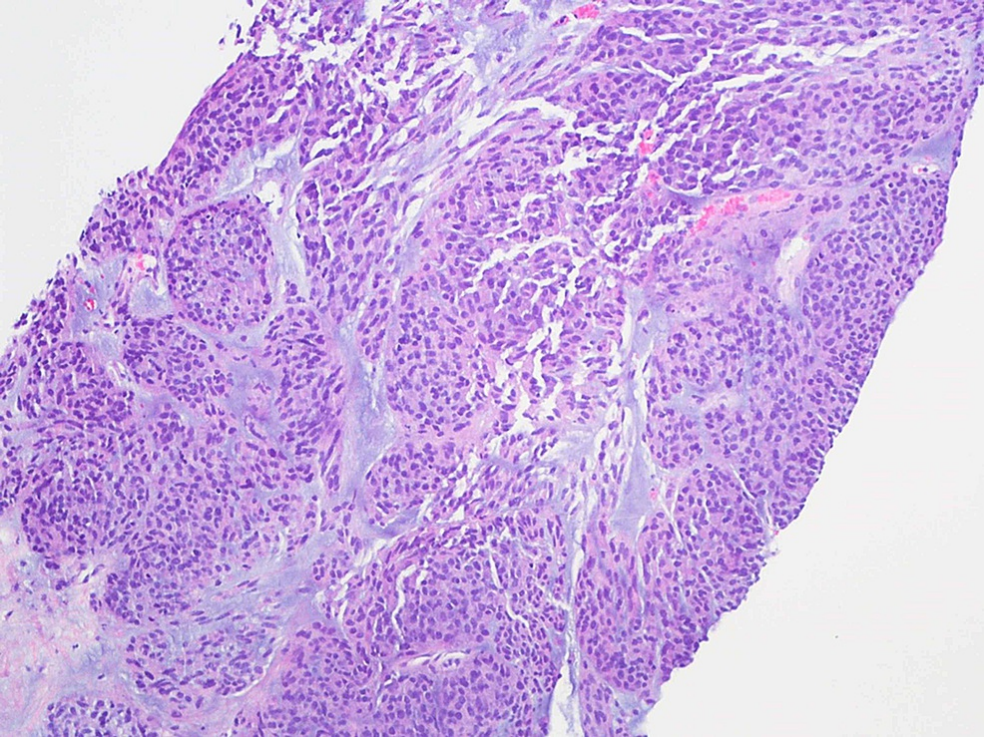
Hematoxylin and eosin (H&E) stain of the mass with magnification x 100 Cellular spindle cell proliferation within loose stroma. The tumor cells are arranged in sheets and nodules.

**Figure 3:**
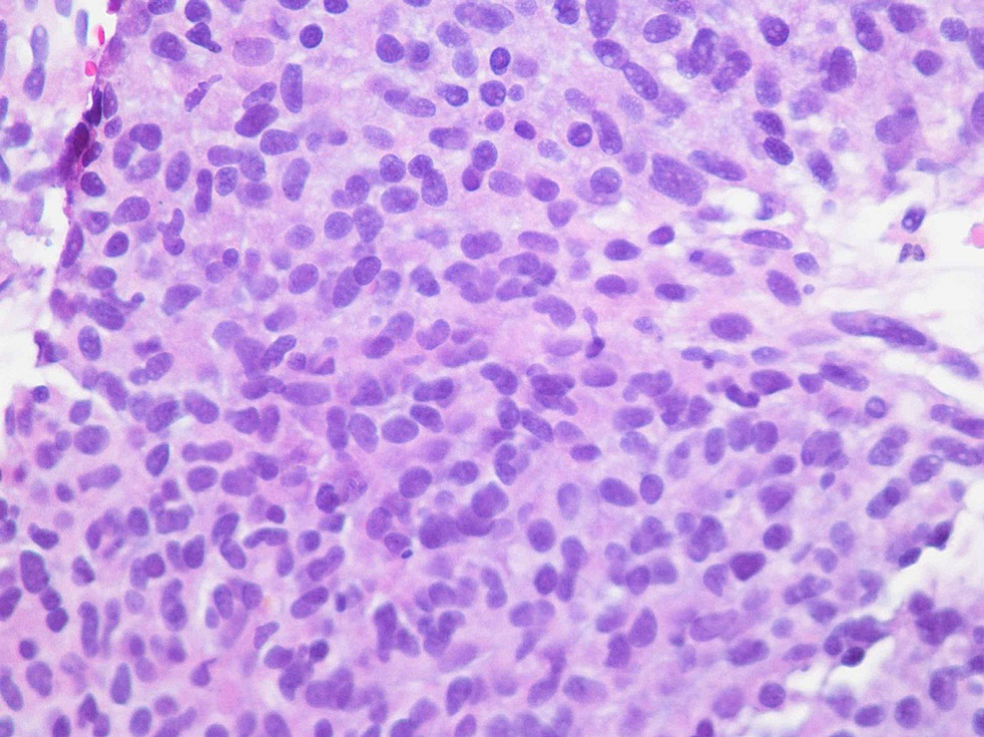
Hematoxylin and eosin (H&E) stain with magnification x 200 Spindle to oval cells with nuclear pleomorphism and arranged in sheets.

**Figure 4:**
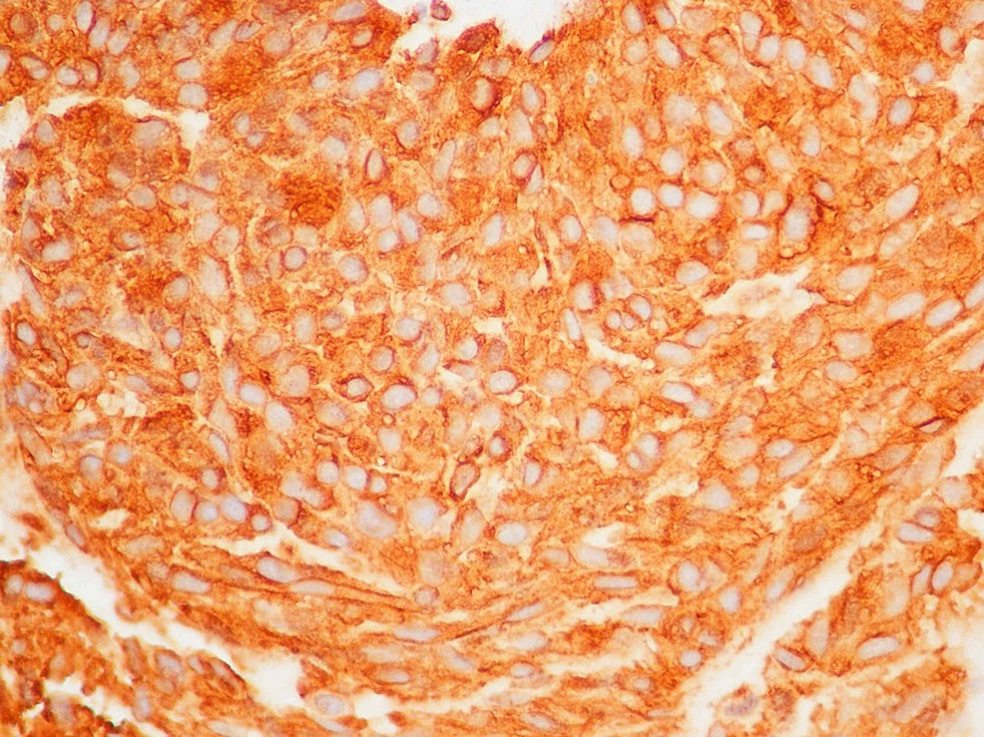
Immunohistochemistry, CD117 The tumor cells are strongly immunoreactive to CD117 (KIT).

A 59-year-old woman presented to her primary care physician with left-sided abdominal pain for the past eight months associated with a weight loss of 20 pounds. She denied any other GI symptoms. She had no history of smoking, alcohol or drug use. On initial examination in the emergency department, the patient was afebrile, hemodynamically stable and was in no acute distress. Her abdomen was soft, non-tender and there was no organomegaly or mass appreciated. The rest of the review of systems and physical examination was unremarkable. CT scan of abdomen with intravenous contrast revealed a 16 x 16 x 15.4 cm lesion with a peripheral solid mass and central hypodensity in the left upper quadrant compressing on the spleen, stomach and liver (Figure 1). There was a 3 mm liver hypodensity. She underwent ultrasound-guided fine-needle aspiration (FNA) biopsy of the lesion and 500 ml of brownish material was aspirated. The histopathological exam of the specimen showed high-grade gastrointestinal stromal tumor (GIST) of spindle cell type with increased mitotic activity (>10/50 high-power field [HPF]). The immunohistochemical stain revealed the tumor cells were positive for cluster of differentiation (CD)34, CD117, DOG1, and smooth muscle antibodies (SMAs) and negative for CK7, CK20, and S100 antibodies. The KI67 index was approximately 20% (Figures 2-4). She was evaluated by the oncology and surgery team. She received neoadjuvant chemotherapy with tyrosine kinase inhibitor (imatinib). Follow up CT scan showed good response and the lesion decreased to 10.8 x 5.0 x 6.0 cm. She underwent partial gastrectomy with resection of the tumor. The pathology specimen confirmed negative margins. She was advised to continue imatinib for a total of three years post-operatively. Three months after surgery she underwent an upper gastrointestinal endoscopy that showed no residual or recurrent tumor.

## Discussion

GISTs are subepithelial tumors, initially referred to as leiomyoma, leiomyosarcoma or leioblastoma. However, with the availability of electron microscopy and immunohistochemical staining, Mazur and Clarks were able to distinguish GISTs as a separate gastrointestinal neoplasm with distinct features [2].

GISTs represent 1% of gastrointestinal malignancies with an incidence of seven per million per year [3]. GISTs originate from CD34-positive stem cells residing within the wall of the gut, which can then differentiate incompletely toward the interstitial cells of Cajal (ICC) phenotype. More than 95% of GISTs exhibit KIT (CD117). Expression of CD34 is not specific for GISTs but is noted to be a poor prognostic indicator as most cases of malignant GIST are CD34 positive. Five percent of GIST cells are not caused through activation and aberrant signaling of the KIT receptor, but rather through mutational activation of the structurally related kinase known as the platelet-derived growth factor receptor- alpha (PDGFRA). Definitive diagnostic criteria for CD117-negative true GIST are currently obscure. The DOG1 gene, which encodes for chloride channel protein actin 1 (independent of KIT and PDGFRA), was discovered in 2004 and is specific for GIST in appropriate clinical and pathological context. Expression of CD117 could be heterogenous, and biopsy could be false negative. True leoisarcomas express smooth muscle actin and desmin but are negative for CD117. Schwannomas are positive for neural antigen S100. GIST tumors are classified into three types based on histopathologic appearance: spindle cell type (70%), epithelioid type (20%) or mixed type (10%) [4-5].

GISTs have a wide variety of clinical presentations depending on the site of involvement. The most common site is the stomach (60-70%), followed by the small intestine (20-30%), colon, rectum (5%), and esophagus (<5%) [6]. Small GISTs may be asymptomatic and are discovered incidentally during endoscopy. Esophageal GISTs may present with dysphagia [7], and stomach or small intestinal GISTs may present with overt or occult gastrointestinal bleeding [8]. Colonic GISTs may present with acute abdomen, perforation, pain or obstruction [9]. Large tumors may present as an abdominal mass or symptoms of paraneoplastic syndrome. Consumptive hypothyroidism caused by marked overexpression of the thyroid hormone-inactivating enzyme type 3 iodothyronine deiodinase (D3) within GISTs has been reported [10]. Malignant GISTs may present with metastasis most commonly to the liver and peritoneum.

The diagnosis of GIST is often suspected on contrast-enhanced CT or magnetic resonance imaging (MRI) showing an abdominal mass. Imaging can also evaluate the extent of the tumor and assess for the presence of metastasis [11]. Endoscopy can be done to evaluate a luminal involvement by the mass. However, an endoscopic ultrasound (EUS) can differentiate intramural and extramural lesions and can further characterize the mass by identifying its layer of origin and allowing ultrasound-directed fine needle aspiration (FNA) biopsy to be obtained for definitive diagnosis. Usually GISTs are well-demarcated, hypoechoic lesions arising from the fourth layer of the gastrointestinal tract (muscularis propria), although small lesions may arise from the second layer (muscular mucosae) [12-13].

GISTs may have different clinical behaviors depending on the site and mitotic activity. In the stomach the benign lesions outnumber the malignant ones, in contrast to the esophagus and colon where most of the GISTs are malignant. Tumors with low mitotic activity, five or fewer mitoses per 50 HPF, usually have a benign behavior as compared to those with more than five per 50 HPF are described as malignant. Tumors with more than 50 mitoses per 50 HPF are described as high-grade malignant [14].

Treatment for GIST depends on the tumor size and location. Esophageal GISTs greater than 2 centimeters need to be excised [15], however for those smaller than 2 centimeters different guidelines recommend different management; there is a conservative approach with follow up with repeat esophagogastroduodenoscopy (EGD) and removal if there is an increase in size. Another approach recommends removal of the tumor for fear of the risk of metastasis [16]. For gastric GISTs, submucosal lesions <1 cm with EUS findings suggestive of benign tumor may be followed conservatively. Management of gastric lesions between 1 and 2 cm is controversial and lesions greater than 2 cm should be excised [17]. For duodenal GISTs, excision is advised whether endoscopically or surgically with pancreaticoduodenectomy [18]. For GISTs in the colon and rectum, excision of the tumor is advised, however sometimes it is challenging especially in the rectum so preoperative imatinib is advised to downsize the tumor and achieve better surgical outcomes [19].

## Conclusions

GISTs comprise only 1% of gastric neoplasms. Clinical features and management depend on the location, size, appearance and characteristics on EUS. Tumors with benign features can be monitored while large tumors should be excised, with neoadjuvant chemotherapy to downsize the tumor prior to resection. Our patient presented with weight loss and abdominal pain, which is a common symptom of many pathologic conditions. It is important to consider GISTs as one of the differential diagnoses when patients present with similar symptoms, as early stage diagnosis improves outcome and long-term prognosis.
